# The Discovery of a Specific CKIP-1 Ligand for the Potential Treatment of Disuse Osteoporosis

**DOI:** 10.3390/ijms25168870

**Published:** 2024-08-15

**Authors:** Yange Wei, Bo Wu, Mingqiu Liu, Chun-Ping Cui

**Affiliations:** State Key Laboratory of Medical Proteomics, National Center for Protein Sciences (Beijing), Beijing Institute of Lifeomics, Beijing 100850, China; weiyange2013@126.com (Y.W.); soldier2158wubo@163.com (B.W.); mingqiurui@163.com (M.L.)

**Keywords:** casein kinase 2 interacting protein-1, proteolysis-targeting chimeras, DNA-encoded libraries, von Hippel–Lindau, disuse osteoporosis

## Abstract

Bone homeostasis relies on the delicate balance between osteoblast-mediated bone formation and osteoclast-mediated bone resorption. The casein kinase 2 interacting protein-1 (CKIP-1), a specific CK2α subunit-interacting protein, has been documented as one of the crucial negative regulators of bone formation. CKIP-1 siRNA therapy has constraints that limit its use in clinical applications. Therefore, it is necessary to explore effective targeting strategies for CKIP-1. In this study, we observed an upregulation of CKIP-1 protein expression in the microgravity environment, while its ubiquitination levels decreased. We further investigated the interaction between CKIP-1 and VHL and found that VHL enhanced CKIP-1 degradation through the ubiquitylation–proteasome system (UPS). Additionally, we discovered a small molecule ligand, named C77, through DNA-encoded library (DEL) screening, which binds to CKIP-1 both in vivo and in vitro, as confirmed by Surface Plasmon Resonance (SPR) and the Cellular Thermal shift assay (CETSA), respectively. Our findings demonstrated the potential of VHL and C77 as guiding factors in the development of CKIP-1-based Proteolysis-Targeting Chimeras (PROTACs), which could be future therapeutic interventions in disuse osteoporosis.

## 1. Introduction

Bone homeostasis depends on the dynamic balance between osteoblast-mediated bone formation and osteoclast-mediated bone resorption [1]. The effects of gravity and mechanical loading are crucial for maintaining bone homeostasis. However, prolonged bed rest or exposure to space flight can lead to bone loss and disuse osteoporosis [2]. This irreversible bone loss caused by mechanical unloading poses a significant health challenge, particularly for astronauts on long-duration space missions [3], and has become one of the most significant limiting factors in long-term orbital spaceflight [4]. While physical exercise and nutritional interventions have proved to mitigate bone loss in astronauts, the currently available effective therapies are limited [5]. Currently, most osteoporosis treatment drugs primarily focus on inhibiting bone resorption rather than promoting bone formation; however, their long-term use may lead to complications such as osteonecrosis and difficulties in replenishing lost bone mass [6]. Therefore, it is necessary to develop an effective treatment to increase bone formation in osteoporosis induced by microgravity.

Casein kinase 2-interacting protein-1 (CKIP-1) functions as a coactivator of Smad ubiquitination regulatory factor 1 (Smurf1) and performs a critical negative role in the regulation of bone formation rather than bone resorption [7]. Accumulated evidence strongly supports CKIP-1 as an attractive therapeutic target in osteoporosis [8,9,10,11,12]. Previous studies have demonstrated that the tissue-specific delivery of small interfering RNA (siRNA) targeting CKIP-1 promotes bone formation and increases bone mass in mice with ovariectomized (OVX)-induced osteoporosis [13]. Through an osteoblast-specific delivery system, CKIP-1 siRNA has increased bone mass for reversing osteoporosis induced by aging [14,15]. Guo et al. demonstrated that CKIP-1 siRNA treatment promotes osteoblast differentiation and improves bone microarchitecture in multiple species, including humans, rhesus monkeys, and rats [16]. These findings highlighted the feasibility of targeting CKIP-1 as a new therapeutic approach for the treatment of osteoporosis [17]. However, the clinical application of CKIP-1 siRNA has been limited because of inherent challenges associated with siRNA technology, including issues related to stability, off-target effects, low specificity, and inadequate drug delivery [18]. Furthermore, studies have shown that CKIP-1-deficient mice can counteract osteoporosis induced by simulated microgravity [19]. Therefore, there is an urgent need to develop next-generation CKIP-1-targeted therapeutics to prevent and treat disuse osteoporosis effectively.

In recent years, Proteolysis-Targeting Chimeras (PROTACs) have emerged as a promising strategy with great potential in drug discovery and biological research [20]. A PROTAC is a heterobifunctional molecule that acts as a degrader, coupling a small molecule ligand (warhead) of the proteins of interest (POI) to an E3 ubiquitin ligase-recruiting moiety through a suitable linker [21]. PROTACs artificially bring the POI close to the E3 ligase, forming a POI-PROTAC-E3 ternary complex that triggers the polyubiquitination modification of the POI, subsequently leading to its recognition and degradation by the 26S proteasome [22]. As a scaffold protein lacking enzymatic activity [23], CKIP-1 seems to be an undruggable target, and the development of selective small molecule inhibitors targeting CKIP-1 has proven to be challenging. Thus, targeting CKIP-1 degradation using the PROTAC technique can be seen as a potential approach.

In this study, we initially demonstrated that the stability of CKIP-1 was increased in both tissues and cell levels induced by microgravity. We revealed that the E3 ligase von Hippel–Lindau (VHL) interacted with and promoted the ubiquitination of CKIP-1. Additionally, we utilized HaloPROTAC3 to demonstrate the ability of the VHL E3 ligase complex to degrade the CKIP-1 protein through the ubiquitin–proteasome pathway (UPS). Furthermore, we reported the discovery of C77 by DNA-encoded libraries (DELs), a nanomolar CKIP-1 ligand. These findings identify VHL and C77 as molecular candidates and provide a theoretical basis for further development of CKIP-1-based PROTAC for the prevention and treatment of disuse osteoporosis.

## 2. Results

### 2.1. CKIP-1 Protein Level Is Upregulated under Microgravity Stimulation

**Figure 1:**
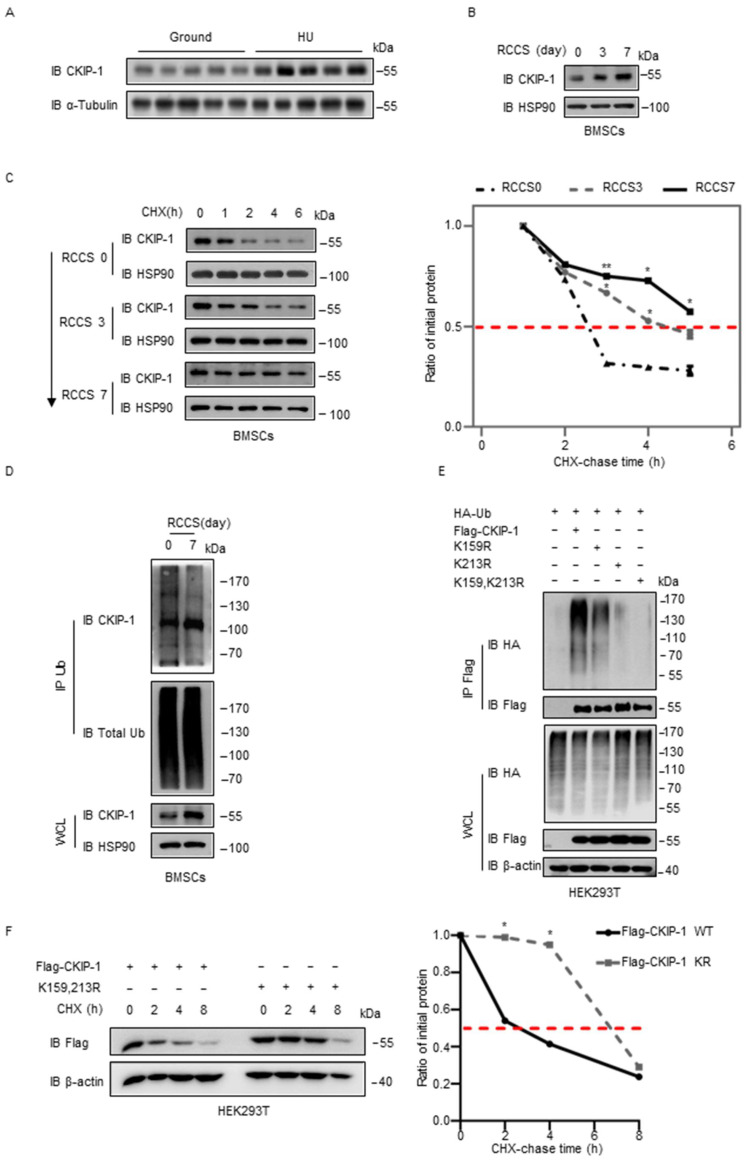
The protein expression of CKIP-1 is upregulated by simulated microgravity and has the potential to be ubiquitinated. (**A**) Immunoblot analysis of CKIP-1 protein levels in femur tissues isolated from WT and HU mice; *n* = 5 per group. (**B**) Immunoblot analysis of CKIP-1 protein levels in BMSCs cultured in RCCS for 3 days or 7 days. (**C**) BMSCs were cultured in RCCS, treated with CHX at different times, and collected at the indicated times for immunoblotting. The quantification of CKIP-1 protein levels relative to HSP90 is shown. (**D**) Immunoblot of CKIP-1 polyubiquitination levels in BMSCs after RCCS for 7 days. (**E**) Immunoprecipitates of CKIP-1 ubiquitination in HEK293T cells transfected with Flag-CKIP-1 or its lysine mutants, HA-tagged WT Ub for 24 h, and then treated for 8 h with MG132 (20 µM), followed by immunoblotting with the indicated antibodies. The K residues in CKIP-1 indicated that lysine residues were mutated to arginine. (**F**) HEK293T cells were transfected for 24 h with Flag-tagged CKIP-1 WT or mutations and then treated with CHX for the indicated time period. The indicated proteins were detected by immunoblotting. The graph shows the quantification of relative CKIP-1 levels. All results are shown as mean ± s.d.; *n* = 3 independent experiments. Statistical analyses in (**C**,**E**) were performed using Student’s *t*-test. * *p* < 0.05, ** *p* < 0.01, compared with control group.

In order to determine the effect of CKIP-1 in weightlessness-induced osteoporosis, we generated the HU mice model. It is worth noting that these mice were subjected to tail suspension and exhibited pronounced bone mass loss after one month (Figure S1A). Furthermore, we observed that the trabecular bones of the HU mice displayed significantly lower BMD, BV/TV, Tb.Th, and Tb.N while exhibiting significantly higher Tb.Sp and BS/BV compared with the control group (Figure S1B). In addition, biomechanical tests revealed a significant reduction in the maximum load and stiffness of the tibia bones in HU mice (Figure S1C). These results indicated a decrease in strength and toughness in HU mice compared with the control group. Consistently, the decreased osteogenesis observed in the HU mice was further validated by a substantial decrease in the mRNA levels of osteoblast differentiation marker genes, such as *Runx2*, *Alp*, *Col1a1*, *Ocn*, and *Osx*. However, the mRNA levels of *Ckip-1* were significantly elevated (Figure S1D). Interestingly, the protein expression levels of CKIP-1 were significantly higher than the control group (Figure 1A). These findings collectively suggested that CKIP-1 might act as an indicator of disuse osteoporosis.

It was previously reported that osteoblast lineage cells, such as BMSCs, can respond to mechanical loading in bone [24]. In our study, we cultured BMSCs in RCCS to simulate the effects of a microgravity environment. We assessed the expression of osteogenic markers by qRT-PCR and observed a significant decrease in the expression of marker genes, such as *Runx2*, *Col1a1*, and *Opn* (Figure S1E). Interestingly, we noted a gradual increase in the protein expression level of CKIP-1 (Figure 1B). To investigate the dynamics of CKIP-1 expression during the microgravity environment provided by RCCS, we treated BMSCs with the protein synthesis inhibitor CHX and observed a significant prolongation in the half-life of CKIP-1 (Figure 1C). The inhibition of the 26S proteasome by MG132 effectively stabilized CKIP-1 (Figure S2A). Furthermore, we observed that CKIP-1 ubiquitylation levels were markedly reduced after RCCS for 7 days (Figure 1D). An in vivo ubiquitination assay demonstrated that ectopic expression of the CKIP-1 protein efficiently undergoes polyubiquitination in BMSCs and MC3T3-E1 cells (Figure S2B–D). Mass spectrometry (MS) data revealed that residues K159 and K213 in CKIP-1 were conjugation sites of ubiquitination (Figure S2B). In light of these findings, we generated K159 and K213 mutant forms of CKIP-1 by substituting these residues with arginine [R]. Interestingly, we observed that the two mutants were unable to undergo ubiquitination (Figure 1E), and the half-life of the mutated CKIP-1 was significantly prolonged upon treatment with CHX (Figure 1F). These results provide evidence that CKIP-1 is degraded through the ubiquitin-dependent pathway.

### 2.2. VHL Interacts with CKIP-1

**Figure 2:**
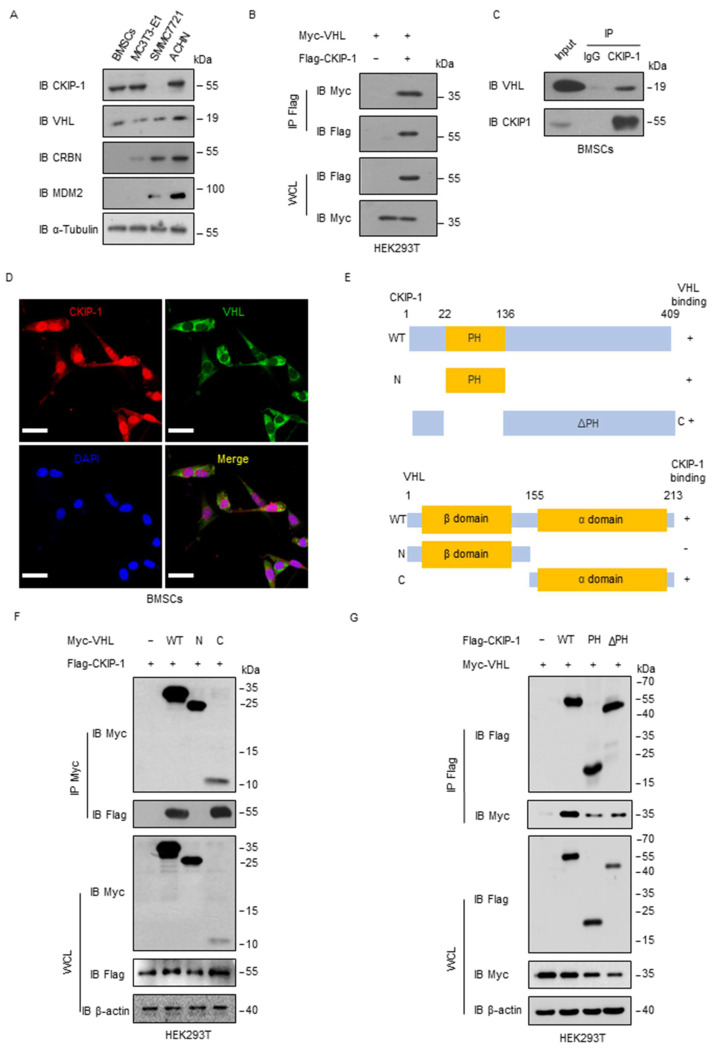
CKIP-1 interacts with VHL. (**A**) The basal protein levels of CKIP-1 and different E3 ligases (VHL, CRBN, and MDM2) commonly used in PROTAC technology were examined using immunoblotting in different cell lines. (**B**) HEK293T cells were transfected for 24 h with Myc-tagged VHL alone or together with Flag-tagged CKIP-1 plasmids and then immunoprecipitated with Flag antibody, followed by immunoblotting with the indicated antibodies. (**C**) BMSC whole-cell lysates were immunoprecipitated with control IgG or CKIP-1 antibody, followed by immunoblotting with the indicated antibodies. (**D**) Immunofluorescence staining was performed by anti-CKIP-1 and anti-VHL antibodies before visualization by confocal microscopy to determine the colocalization of these two proteins in BMSCs (scale bar: 10 µm). (**E**) Overview structure of the WT CKIP-1 and its indicated constructs PH (22–136) and ΔPH (1–22 and 136–409); WT VHL and its indicated constructs. (**F**) HEK293T cells were transfected for 24 h with Myc-tagged VHL alone or together with Flag-tagged WT CKIP-1 or its indicated constructs and then immunoprecipitated with Flag antibody, followed by immunoblotting with the indicated antibodies. (**G**) HEK293T cells were transfected for 24 h with Flag-tagged WT CKIP-1 alone or together with Myc-tagged VHL and then immunoprecipitated with Myc antibody, followed by immunoblotting with indicated antibodies. *n* = 3 independent experiments.

Considering that the CKIP-1 protein level was upregulated in response to microgravity stimulation, we proposed the use of PROTAC technology to target its degradation as a potential treatment for disuse osteoporosis. To assess the feasibility of this approach, we examined the expression of E3 ligases commonly employed in PROTACs in different cell lines. We observed higher levels of VHL expression in osteoblasts, BMSCs, and MC3T3-E1 compared with the expression levels of cereblon (CRBN) or mouse double minute 2 homolog (MDM2) (Figure 2A). Interestingly, the protein expression level of VHL was also increased in BMSCs after culturing with RCCS (Figure S3A). Co-immunoprecipitation (Co-IP) experiments revealed the interaction between exogenous CKIP-1 and VHL in HEK293T cells (Figure 2B), which was further validated in BMSCs (Figure 2C). The immunofluorescence (IF) assay revealed the colocalization of CKIP-1 and VHL in the cytoplasm region (Figure 2D). It is worth noting that CKIP-1 possesses a pleckstrin homology (PH) domain at the N-terminus and a leucine zipper (LZ) motif at the C-terminus. The VHL protein comprises two distinct domains including a large NH2-terminal domain rich in β sheets (β-domain) and a smaller α-helical domain (α-domain), which are connected by two linkers and a polar interface [25]. To determine the specific interaction domain (s) between CKIP-1 and VHL, we generated three deletion mutants (Figure 2E). Co-IP assays revealed that the C-terminal α-domain of VHL, rather than the N-terminal β-domain, was responsible for the physical interaction with CKIP-1 (Figure 2F). Furthermore, both the PH domain and the ΔPH domain of CKIP-1 were found to interact with VHL (Figure 2G). These findings indicated that the interaction between CKIP-1 and VHL was mediated by the C-terminal α-domain of VHL.

### 2.3. VHL Promotes Ubiquitylation of CKIP-1

**Figure 3:**
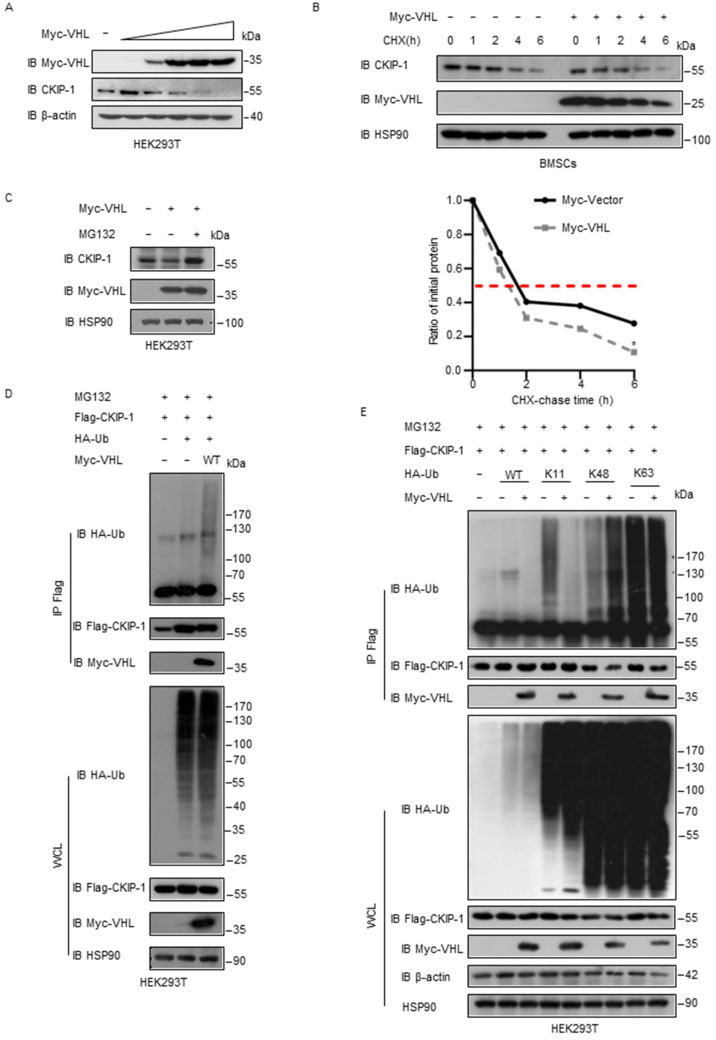
VHL downregulates CKIP-1 levels and promotes its ubiquitylation. (**A**) HEK293T cells transfected with increasing amounts of Myc-tagged VHL plasmids were collected 24 h post-transfection. The indicated proteins were detected by immunoblotting with the indicated antibodies. Results are shown as mean ± s.d. (**B**) BMSCs transfected with Myc-tagged VHL and then treated with CHX for the indicated time period. The indicated proteins were detected by immunoblotting. The quantification of relative CKIP-1 levels is shown. Results are shown as mean ± s.d. Statistical analyses were performed using Student’s *t*-test. * *p* < 0.05, compared with control group. (**C**) HEK293T cells were transfected for 24 h with or without Myc-tagged VHL plasmids, and then treated with or without MG132 (20 µM) for 8 h. The indicated proteins were detected by western blotting. (**D**) HEK293T cells were transfected for 24 h with Flag-tagged CKIP-1, HA-tagged Ub, and Myc-tagged VHL and then treated for 8 h with MG132 (20 µM). Cell lysates were immunoprecipitated with Flag antibody, followed by immunoblotting with indicated antibodies. (**E**) HEK293T cells were transfected for 24 h with Flag-tagged CKIP-1, Myc-tagged VHL, HA-tagged WT Ub, or mutant Ub (K11, Lys 11-only; K48, Lys 48-only; K63, Lys 63-only) alone or in combination, and then treated for 8 h with MG132 (20 µM). Cell lysates were immunoprecipitated with Flag antibody, followed by immunoblotting with indicated antibodies. *n* = 3 independent experiments.

Given that VHL functions as a ubiquitin ligase, we hypothesized that it might serve as a negative regulator for CKIP-1 levels. To test this hypothesis, we ectopically expressed VHL and observed a dose-dependent downregulation of the CKIP-1 level both exogenously and endogenously (Figure 3A and Figure S3B). Subsequently, we revealed that the half-life of CKIP-1 was reduced in cells that overexpressed VHL (Figure 3B and Figure S3C), demonstrating that VHL promotes the degradation of CKIP-1 to downregulate its levels. Moreover, we examined whether this degradation of CKIP-1 is dependent on proteasomes. Indeed, the proteasome inhibitor MG132 prevented the downregulation of CKIP-1 induced by VHL (Figure 3C). In order to investigate the mechanism of CKIP-1 decrease by VHL, we examined whether VHL promotes CKIP-1 ubiquitylation. We found that VHL indeed enhanced the ubiquitylation of CKIP-1 in HEK293T cells (Figure 3D). Furthermore, ubiquitination assays revealed that VHL specifically promoted Lys 48-linked ubiquitylation of CKIP-1 while having no effect on nondegradative Lys 63-linked ubiquitylation (Figure 3E and Figure S3D). These findings collectively indicated that VHL may serve as a ubiquitin E3 ligase for CKIP-1, leading to its ubiquitylation and destabilization.

### 2.4. HaloPROTAC3 Degrades CKIP-1

**Figure 4:**
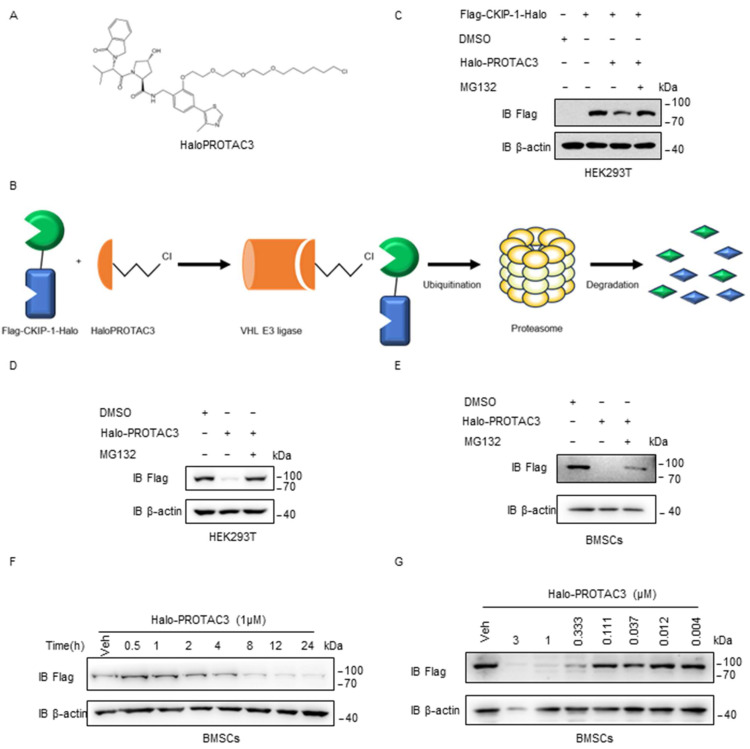
HaloPROTAC3 can degrade CKIP-1 in osteoblasts. (**A**) The chemical structure of HaloPROTAC3. (**B**) Schematic of HaloPROTAC3 degradation of HaloTag protein fusions in live cells. HaloPROTAC3 induces a ternary complex between the VHL E3 ligase component and HaloTag protein fusion, resulting in the degradation of the HaloTag target protein via the ubiquitin–proteasomal pathway. (**C**) HEK293T cells were transfected for 24 h with Flag-CKIP-1-Halo plasmids and then the cells were treated with or without HaloPROTAC3 (1 μM) and MG132 as indicated for 12 h, followed by immunoblotting with indicated antibodies. (**D**) A representative immunoblot analysis showing that HaloPROTAC3 induces Flag-CKIP-1-Halo degradation in a proteasome-dependent manner. Flag-CKIP-1-Halo was packaged into lentiviral particles and transduced into HEK293T cells. The positive clones were selected by puromycin (0.5 µg/mL). (**E**) A representative immunoblot analysis showing that HaloPROTAC3 induces Flag-CKIP-1-Halo degradation in a proteasome-dependent manner. Flag-CKIP-1-Halo were packaged into lentiviral particles and transduced into BMSCs. The positive clones were selected by puromycin (3 µg/mL). (**F**) HaloPROTAC3 degrades Flag-CKIP-1-Halo in BMSCs after treatment with vehicle (Veh) or increasing concentrations of HaloPROTAC3 as indicated for 12 h, followed by immunoblotting with indicated antibodies. (**G**) Immunoblot analysis of Flag-CKIP-1-Halo expression in BMSCs after they were treated with HaloPROTAC3 for various durations as indicated. *n* = 3 independent experiments.

Previous studies have indicated that the regulation of target protein loss can be achieved using HaloPROTAC3 (Figure 4A), which selectively degrades HaloTag fusion proteins by recruiting the specific VHL E3 ligase component [26]. The schematic diagram in Figure 4B illustrates the degradation of the fusion protein Flag-CKIP-1-Halo in live cells by HaloPROTAC3. In brief, HaloPROTAC3 promoted the assembly of a ternary complex comprising the VHL E3 ligase component and Flag-CKIP-1-Halo, which subsequently underwent degradation via UPS. With HaloPROTAC3, we investigated the potential use of VHL as an E3 ligase in designing PROTACs targeting CKIP-1. Treatment with 1 μM HaloPROTAC3 resulted in the nearly complete knockdown of Flag-CKIP-1-Halo, as illustrated in Figure 4C,D. Immunoblotting assays further confirmed the results in BMSCs, as depicted in Figure 4E. In addition, time- and concentration-dependent experiments were conducted to further validate the degradation capability of HaloPROTAC3 in BMSCs (Figure 4F,G). Based on the results, VHL can be chosen as the E3 ligase for designing PROTACs to degrade CKIP-1 in BMSCs.

### 2.5. Hit Generation from DELs Screening Data

**Figure 5:**
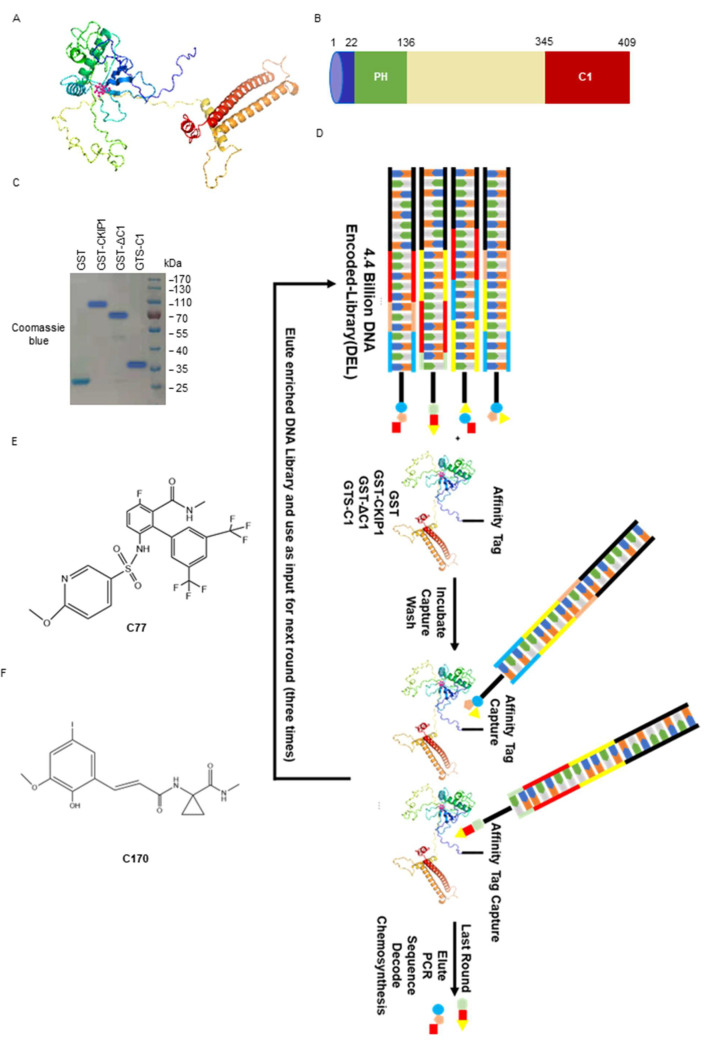
The CKIP-1 ligand was obtained through DEL screening. (**A**) Predicting the structure of protein CKIP-1 using AlphaFold. (**B**) The structural pattern of protein CKIP-1. (**C**) Expression of purified prokaryotic protein GST, GST-CKIP-1, GST-ΔC1, and GST-C1. (**D**) Schematic diagram of the DEL screening. DELs were incubated with GST, GST-CKIP-1, GST-ΔC1, and GST-C1, affinity captured, washed to remove the non-binding library, and then enriched library members were eluted. After repeating three times, the DNA tags were sequenced to identify the small molecule ligands. (**E**) The chemical structure of compound C77. (**F**) The chemical structure of compound C170.

In our previous experiments, it was observed that the purified CKIP-1 protein is unstable, and it is difficult to resolve its crystal structure [27]. We utilized AlphaFold (2.0, Google DeepMind, London, UK) (https://alphafold.ebi.ac.uk/entry/Q53GL0), accessed on 8 October 2021, to predict the protein structure of CKIP-1, as depicted in Figure 5A. Unfortunately, virtual screening did not yield any ligands for CKIP-1 because of the absence of a pocket active region. To overcome this challenge and discover novel ligands for CKIP-1, an effective strategy, DNA-encoded libraries (DELs), was employed for screening. We analyzed the sequence and predicted structure of CKIP-1 before screening, as shown in Figure 5B. CKIP-1 consists of a pleckstrin homology (PH) domain (22–136) at the N-terminus, a putative leucine zipper (LZ) motif at the C-terminus (designated as C1, 345–409), and five proline-rich motifs distributed throughout the protein. It is worth noting that though the PH domain of CKIP-1 adopts a stable conformation, there is a risk of being off-target because of numerous other proteins (up to 252) in the cell that also contain PH domains. The intermediate region (137–344) is a long and flexible region, which includes the two lysine residues (K159, K213) associated with ubiquitination degradation. Therefore, the binding of compounds to this region may influence the CKIP-1 ubiquitination degradation process. The carboxyl end of CKIP-1 (CKIP1-C1) contains a rigid leucine zipper (LZ) zone, which can function independently and is suitable for potential binder screening. Taking these factors into consideration, we purified GST-CKIP-1, GST-CKIP-1-ΔC1, and GST-CKIP-1-C1 prokaryotic proteins for the screening assay (Figure 5C).

A typical filtering process for DELs is illustrated in Figure 5D. Specifically, DELs are co-incubated with the GST-tagged CKIP-1 protein, and non-affinity fractions are subsequently washed away. The remaining affinity molecules can be separated from the protein by heating and re-entering the solution three times. The small molecule structures exhibiting affinity for target proteins are obtained after PCR amplification, high-throughput sequencing, and data analysis. The flowchart of the DEL screening experimental procedure details is displayed in the Supplementary Materials. Furthermore, based on the evaluation of the enrichment ratio and copy number, the off-DNA hits were synthesized using traditional organic chemical methods by the company (WuXi AppTec, Nanjing, China). In this study, the compounds 10172-10207-57-23-170-0 (C170) (Figure 5E) and 10172-10051-15-479-0-77 (C77) (Figure 5F) were synthesized as candidate ligands because of their highest enrichment ratio and copy number compared with other ligands. Collectively, these findings indicate the identification of two compounds capable of binding to CKIP-1 through DEL screening.

### 2.6. Biophysical Characterization of Compounds with CKIP-1

**Figure 6:**
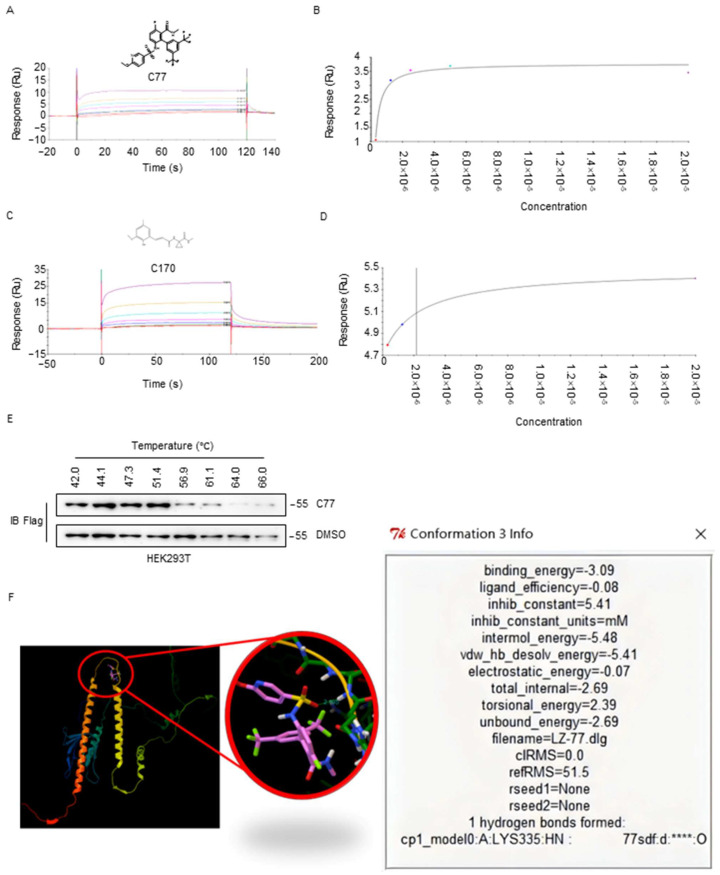
Biophysical characterization of compound C77 with CKIP-1. (**A**) High-density sensorgrams of representative SPR for CKIP-1-C1 by compound C77. (**B**) Immobilization steady-state fit of the dose–response curve of compound C77. Low density, 1000 RU, KD: 27.12 nM. (**C**) High-density sensorgrams of representative SPR for CKIP-1-C1 by compound C170. (**D**) Immobilization steady-state fit of the dose–response curve of compound C170. Low density, 1000 RU, KD: 2.169 μM. (**E**) CETSA for Flag-CKIP-1. HEK293T cells with stable expression of Flag-CKIP-1 were treated with DMSO or 1 μM of C77 for 6 h. (**F**) The virtual docking of CKIP-1 (represent with ****) and compound C77 using AutoDock software 4.0.

Two selected compounds (C77 and C170) were examined by biophysical and biochemical assays. The binding affinity and target engagement in vitro were assessed by Surface Plasmon Resonance (SPR). The results showed that C77 bound to GST-CKIP-1-C1 with high potency, exhibiting a KD value of 27.12 nM, while C170 displayed potent binding to CKIP-1-C1, with a KD of 2.169 μM (Figure 6A–D). Moreover, to confirm the target engagement of C77 (Supplementary Data) in vivo, the Cellular Thermal shift assay (CETSA) was performed in HEK293T cells with stable Flag-CKIP-1 expression. Immunoblotting assay revealed that Flag-CKIP-1 could be stabilized significantly (denaturation temperature > 2 °C) by C77 exposure in HEK293T cells (Figure 6E). Furthermore, the binding analysis between CKIP-1 and C77 was predicted by AutoDock software 4.0. The hydrogen bonds were formed in amino acid position 335 with a binding energy of 3.09 (Figure 6F). These findings suggest that C77 exhibits a higher affinity towards CKIP-1 and holds the potential for PROTAC design.

## 3. Discussion

The elimination of CKIP-1 at the protein level is of utmost importance in the development of novel therapeutic approaches for the prevention and treatment of disuse osteoporosis. In this study, our findings indicated that the protein expression level of CKIP-1 is upregulated, while its ubiquitination is decreased in BMSCs exposed to a microgravity environment. Additionally, we revealed a previously unrecognized interaction between CKIP-1 and VHL. It is worth noting that VHL is an integral component in the chemical toolbox for PROTAC design [28]. Lastly, we reported the discovery of a small molecule, C77, that targets CKIP-1 through DEL screening. These results provide a solid basis for the use of PROTAC technology to degrade CKIP-1 for the prevention and treatment of disuse osteoporosis.

Bone mass in adults is maintained through a delicate balance between bone resorption and bone formation [29]. In fact, astronauts experience a loss of approximately 2% of mineral density during one month of microgravity-induced bone loss [30]. Numerous studies suggest that the suppression of bone formation is primarily responsible for the bone loss observed in microgravity conditions [31], yet effective methods to counteract this phenomenon are currently lacking. The transforming growth factor-beta and bone morphogenic protein (TGF-β/BMP) signaling pathway plays a crucial role in bone formation [32]. CKIP-1 negatively regulates the BMP pathway, leading to a reduction in bone formation without significantly impacting bone resorption [7,33]. Targeting CKIP-1 represents an appealing therapeutic strategy for osteoporosis treatment. Various studies have unveiled the vital role of CKIP-1 in maintaining bone homeostasis through gene downregulation [13,15,17]. However, relying on siRNA technology to reduce CKIP-1 gene expression for the clinical treatment of osteoporosis is not feasible. In this study, we first investigated the protein expression of CKIP-1, which was found to be upregulated in both femur tissue from HU mice and BMSCs cultured in RCCS. These findings highlight CKIP-1 at the protein level as an attractive target for drug discovery. Targeted protein degradation by hijacking the ubiquitin–proteasome system has emerged as a unique strategy in drug discovery across therapeutic areas [34]. Therefore, utilizing PROTAC technology to degrade CKIP-1 presents a viable approach for the prevention and treatment of disuse osteoporosis.

PROTACs utilize E3 ligase to induce protein degradation. If E3 ligase is cell- or tissue-specific; even if the target protein is expressed ubiquitously, the degradation of the target protein by PROTACs can achieve cell or tissue selectivity [35,36]. In comparison with CRBN and MDM2, VHL demonstrates maximal expression in osteoblasts. This indicates that the expression of VHL is cell-selective, particularly in osteoblasts. In order to achieve degradation of the target protein by PROTACs, the key step is the formation of a ternary complex involving the target protein, a PROTAC, and the E3 ubiquitin ligase enzyme [37]. Previous studies have shown that favorable interactions between the target protein and E3 ligase, resulting in positive cooperativity (α > 1), can stabilize the formation of the ternary complex [38]. In other words, the stabilized target–ligase protein–protein interactions (PPIs) may be a powerful strategy in designing efficient PROTACs [39]. In this study, several lines of evidence support the conclusion that CKIP-1 interacts with VHL. Firstly, Co-IP assays demonstrated the endogenous binding of CKIP-1 to VHL in BMSCs. Secondly, CKIP-1 and VHL were colocalized in the cytoplasm by IF in BMSCs. Thirdly, the successful binding of HaloPROTAC3 to Flag-CKIP-1-Halo and VHL was also demonstrated in BMSCs. Collectively, this funneled selection provided compelling evidence for the feasibility of using VHL as part of the PROTAC design for CKIP-1.

Additionally, we discovered that CKIP-1, as a negative regulator of bone formation, could be ubiquitylated and undergo proteasomal degradation. Intriguingly, our hypothesis suggested that VHL could potentially act as the E3 ligase responsible for CKIP-1 ubiquitylation. Through our experiments, we confirmed that VHL negatively regulates CKIP-1 levels. Overexpression of VHL resulted in increased CKIP-1 ubiquitylation, as validated by in vitro ubiquitylation assays. Notably, VHL primarily promoted Lys 48-linked ubiquitylation of CKIP-1, while non-degradative Lys 63-linked ubiquitylation was not significantly affected. However, further investigations are needed to explore whether VHL promotes other types of CKIP-1 ubiquitylation, such as Lys 11-linked ubiquitylation. While exogenous overexpression of VHL led to a shortened half-life of CKIP-1 in BMSCs, these potential post-translational modifications warrant future research into the feasibility of degrading CKIP-1 using PROTAC technology. In a previous study, it was highlighted that the E3 ligase c-Cbl mediates CKIP-1 ubiquitination to downregulate CKIP-1 protein expression in diabetic kidneys [40]. Therefore, the specificity of VHL as the E3 ligase for CKIP-1 in osteoblasts remains to be determined in the subsequent steps of our research.

CKIP-1 undruggable disease-causing proteins that lack enzymatic activity are ideal PROTAC targets. Our objective was to identify small molecule ligands for CKIP-1 that could aid in understanding its cellular roles and facilitate the development of PROTACs. DEL technology, which enables the screening of billions of molecules in a single experiment [41], has emerged as a popular approach in drug discovery. Among the DELs screened, only C77 demonstrated the most promising ligand efficiency, as evaluated by the SPR assay with a binding affinity in the nM range. Target engagement of C77 with CKIP-1 in cells was corroborated through CETSA experiments. Molecular docking was performed using AutoDock software 4.0 to simulate a hydrogen bond interaction between C77 and CKIP-1 at Lys 335. Furthermore, after optimizing the physicochemical properties of C77, a library of CKIP-1 PROTAC was constructed by combining the optimized C77 compound with commercially available VHL ligands and various linkers. The surfaces of the POI, E3 ligase, and PROTAC must properly cluster together to form ternary complexes, and this interaction is responsible for triggering ubiquitination. Therefore, our study undoubtedly provides a crucial theoretical basis for the synthesis of highly efficient CKIP-1 PROTACs.

CKIP-1 has many effects on the human body [23]. Previous studies indicate that pathological cardiac hypertrophy worsened in cardiac-specific CKIP-1 KO mutants under cardiac pressure overload, with CKIP-1 overexpression providing protective effects [42]. Additionally, microgravity-induced cardiac atrophy was inhibited in CKIP-1 transgenic mice [43]. Thus, we sought to develop an osteoblast-specific delivery system for osteogenic CKIP-1-PROTAC to address the potential adverse effects. We will use the aptamer CH6 conjugated to lipid nanoparticles (LNPs) encapsulating CKIP-1-PROTAC to specifically target the degradation of CKIP-1 protein in osteoblasts. CH6 is an aptamer selected by using cell-based systematic evolution of ligands exponential enrichment (cell-SELEX), specifically targeting both rat and human osteoblasts [15]. Our next step is to validate the biological function and therapeutic potential of new synthesized CH6-LNPs-CKIP-1-PROTAC within osteoblasts, further exploring its potential for the prevention and treatment of osteoporosis induced by microgravity.

PROTACs may represent a powerful tool to extend druggable space to new target types previously considered intractable or undruggable [44]. According to the latest statistics, PROTAC technology has received great attention in the industry and has been applied to the possible treatment of cancers, immune disorders, viral infections, neurodegenerative diseases, etc. [45]. However, no studies have used the PROTAC technique to prevent or treat osteoporosis. Our study provides a novel perspective on the treatment of disuse osteoporosis through the degradation of CKIP-1. In this study, we demonstrated the interaction between CKIP-1 and VHL and identified VHL as a suitable E3 ligase for the assembly of PROTAC that exhibits cellular selectivity. Our study also led to the discovery of a highly potent and novel molecule ligand, C77, that effectively targets CKIP-1. In conclusion, these findings provide valuable insights for using PROTAC technology to target the degradation of CKIP-1, with potential applications in the treatment of disuse osteoporosis.

## 4. Materials and Methods

### 4.1. Animals Model

A total of 10 two-month-old WT C57BL/6J mice were randomly divided into two groups. HU in mice is a well-established model used to simulate weightlessness-induced bone loss. The HU-induced model mice (*n* = 5) were suspended by their tails to keep their hind legs off the ground. The trunk of the mice was angled at about 30° with respect to the ground, allowing them to move freely using their forelimbs, which was maintained for 4 weeks to induce bone loss. Control mice were not tail-suspended and were allowed to move normally on the ground. The experimental conditions included a room temperature of 24 ± 1 °C, a relative humidity of 60 ± 10%, and a daily light cycle of 10–12 h.

### 4.2. Rotary Cell Culture System (RCCS)

A three-dimensional (3D) rotary cell culture system (Synthecon, Houston, TX, USA) was utilized to simulate the effects of microgravity as described in previous studies [46]. For BMSCs, the cell-density seeding was approximately 1 × 10^6^ cells/mL. The medium was refreshed twice a week. In the case of anchorage-dependent cells with microcarrier beads, the rotation of the vessel began at a speed of about 11 rpm and gradually increased as the multicellular aggregates grew in size, thereby maintaining the aggregates in a constant equilibrium under free-fall conditions. At specific time points, the cells were harvested according to the experimental requirements using 3D FloTrix™ Digest lysate (CytoNiche Biotech, Beijing, China). Then, 3D TableTrix Microcarrier™ (CytoNiche Biotech, Beijing, China) was prepared with serum-free culture medium for cell attachment or stored at 4 °C. BMSCs without rotation were cultured in the same manner as the control group.

### 4.3. Quantitative Real-Time PCR Analysis

Total RNA was extracted from cells or bone tissues using TRIzol Reagent (Thermo Fisher Scientific, Waltham, MA, USA) following the manufacturer’s instructions. Complementary DNA (cDNA) was synthesized from 1 microgram of total RNA using ReverTra Ace^™^ qPCR RT Master Mix (TOYOBO, Osaka, Japan). The cDNA was then used as a template by specific primers for detecting mRNA expression via RT-PCR on an instrument (LightCycler R 96, Roche, Basel, Switzerland) using 2X RealStar SYBR Mixture (Genstar, Beijing, China). The relative gene expression, normalized to GAPDH, was calculated using the 2^−ΔΔCT^ method. The primer sequences used in this study are listed in Supplementary Table S1.

### 4.4. Immunofluorescence

For the detection of subcellular localization by immunofluorescence, BMSCs were fixed in 4% paraformaldehyde for 1 h and then permeabilized with 0.5% Triton X-100 in PBST (0.1% Triton X-100 in PBS) for 15 min. The cells were blocked with 10% goat serum for 1 h at 37 °C. Next, the cells were incubated overnight at 4 °C with primary antibodies against CKIP-1 and VHL. After washing three times with PBST, the cells were stained with secondary antibodies conjugated to Alexa Fluor 488 or 594 (Zhongshan Golden Bridge, Beijing, China) at a ratio of 1:500 in PBS for 1 h at room temperature in the dark. Nuclear staining was performed using the dye 4,6-diamidino-2-phenylindole (DAPI, Sigma-Aldrich, Saint Louis, MO, USA) for 5 min at room temperature in the dark. Following another round of washes, the fluorescence images were visualized using a LSM 510 Meta inverted confocal microscope (Zeiss, Oberkochen, Baden-württemberg, Germany).

### 4.5. Immunoblotting

Total protein concentrations in cell lysates were determined using a BCA kit (Solarbio, Beijing, China) and then further separated on 8–15% SDS-PAGE gels and transferred onto NC membranes (Merck Millipore, Bedford, MA, USA). The membranes were then blocked before overnight incubation with the indicated antibodies (Supplementary Data) at 4 °C. After incubation with the appropriate HRP-conjugated secondary antibodies, the protein bands were visualized using the Super Signal West Pico PLUS chemiluminescent Detection Reagent (Thermo Fisher Scientific, Waltham, MA, USA). The band intensity was quantified with ImageJ 1 software (National Institutes of Health, Bethesda, MD, USA). Statistical analyses of densitometry values were performed with GraphPad Prism 8.0 software (GraphPad Software, San Diego, CA, USA).

### 4.6. Plasmid Constructs

The plasmids encoding human CKIP-1 were previously described [47,48]. Full-length CKIP-1, as well as its truncations, were constructed by PCR and subsequently subcloned into various vectors. For more detailed information, please contact the authors directly. Human CKIP-1 K159R and CKIP-1 K213R were generated using the Gibson Assembly^®^ strategy (Thermo Fisher Scientific, Waltham, MA, USA). The wild-type human VHL, along with its truncations (N-terminal β-domain and C-terminal α-domain), were maintained in our laboratory [49]. Haemagglutinin (HA)-tagged ubiquitin and mouse VHL WT were kindly provided by Wu Bo (Beijing Institute of LifeOmics, Beijing, China).

### 4.7. Mass Spectrometry

HEK293T cells were transiently transfected with plasmids encoding Flag-CKIP-1. The cells were lysed using RIPA buffer (50 mM Tris (pH 7.5), 150 mM NaCl, 1% NP-40, 10 mM NaF, and 1 mM Na_3_VO_4_) containing protease inhibitors (MCE, Monmouth Junction, NJ, USA) and phosphatase inhibitors (Solarbio, Beijing, China). The lysates were then incubated with anti-Flag M2 beads (Sigma-Aldrich, Saint Louis, MO, USA) to capture the Flag-CKIP-1 protein. The bound material was subsequently eluted using 0.1 M glycine-HCl (pH 2.7) and separated by electrophoresis on an 8% gel. The gel was stained with Bio-Safe Coomassie dye to visualize the protein bands. Finally, the eluted protein samples were subjected to mass spectrometry analysis by PTM-Biolabs Co., Ltd. (Hangzhou, China).

### 4.8. Cell Transfection and Immunoprecipitation

HEK293T cells were transfected with the corresponding expression plasmids using LipoPlus (Genstar, Beijing, China). After 24 h, the cells were lysed with HEPES lysis buffer (20 mM HEPES (pH 7.2), 50 mM NaCl, 0.5% Triton X-100, 1 mM NaF, and 1 mM dithiothreitol) containing protease inhibitors. The lysates were then centrifuged at 12,000× *g* for 10 min at 4 °C, and the resulting supernatant was incubated with the corresponding primary antibody and protein A/G agarose beads (Santa Cruz, Dallas, CA, USA) overnight at 4 °C. The beads were washed three times with HEPES buffer and subsequently boiled for immunoblotting. The results were measured and analyzed using ImageJ 1 software (National Institutes of Health, Bethesda, MD, USA).

### 4.9. In Vivo Ubiquitylation Assays

For in vivo ubiquitylation assays, HEK293T cells were transfected with Flag-CKIP-1, Myc-VHL, and WT, K11-linked, K48-linked, or K63-linked HA-Ub with LipoPlus for 24 h. Subsequently, the cells were treated with MG132 for 8 h. The cells were washed with PBS, pelleted, and lysed in RIPA lysis buffer supplemented with protease inhibitor and phosphatase inhibitor. The lysates were incubated with the anti-Flag antibody for 3 h and protein A/G agarose beads for a further 6 h at 4 °C. Afterward, the beads were washed three times with RIPA buffer to remove non-specific binding. The proteins bound to the beads were released by boiling in SDS-PAGE sample buffer and analyzed by immunoblotting using indicated antibodies.

### 4.10. Surface Plasmon Resonance (SPR)

Compound-binding experiments were conducted on a Biacore T200 instrument (Cytiva, Eysins, Switzerland) using a protein–ligand Direct Binding Assay (DBA) at room temperature. The GST antibody was immobilized onto a carboxymethylated dextran surface (CM5) via direct amination, and subsequently, GST-CKIP-1-C1 was captured onto the CM5 surface, serving as the fixed phase. For SPR analysis, C77 and C170 were diluted in 5% DMSO running buffer, serving as the mobile phase. The affinity of each small molecule was determined through repeated experiments. Kinetic curve fittings and KD calculations were performed using a 1:1 binding model and Biacore T200 Evaluation software (Cytiva, Eysins, Switzerland).

### 4.11. Cellular Thermal Shift Assay (CETSA)

The CETSA assay was adapted following the described protocol [35]. Briefly, HEK293T cells overexpressing stable Flag-CKIP-1 were treated with either 1 μM C77 or DMSO for 12 h. Following treatment, the cells were harvested and washed with ice-cold 1X PBS. The cell pellet was then resuspended in PBS containing protease and phosphatase inhibitors. To ensure consistent thawing, the cell lysate was snap-frozen using liquid nitrogen and briefly vortexed. This freeze–thaw process was repeated four times. The soluble fraction was obtained by centrifugation at 20,000× *g* for 30 min at 4 °C and divided into two equal aliquots. Each aliquot (50 µL) was further divided into eight PCR tubes. The samples were individually heated at temperatures ranging from 42 °C to 66 °C for 3 min, followed by cooling at room temperature for 3 min. After centrifugation at 20,000× *g* for 20 min at 4 °C, the soluble fraction was analyzed by immunoblotting.

### 4.12. Micro-Computed Tomography (Micro-CT) Analysis

The bone phenotype of the WT and HU mice on the femur was analyzed by a micro-CT system. The femurs were fixed in 4% polyfluoroalkoxy (PFA) for 48 h and then stored in 70% ethanol at 4 °C before scanning. Images of the femurs of control and osteoporotic mice were scanned using the Inveon MM system (Siemens, Berlin, Germany), and the related parameters were set as described previously [50]. Three-dimensional reconstruction of trabecular was generated from the lowest end of the growth plate extended by 0.5 mm using COBRA software (Exxim Computing Corporation, Pleasanton, CA, USA). Trabecular parameters including bone mineral density (BMD), bone volume/bone total volume (BV/TV), trabecular thickness (Tb.Th), number of trabeculae (Tb.N), trabecular spacing (Tb.Sp), and bone surface/bone volume (BS/BV) were assessed using Inveon Research Workplace analysis software (Siemens, Berlin, Germany).

### 4.13. Mechanical Properties

Immediately after dissection, the biomechanical properties of the tibia were subjected to a three-point bending analysis using a CellScale Biomaterials testing device (CellScale, Toronto, ON, Canada). Experiments were performed as described previously [51]. Biomechanical measurement data were collected from the load–deformation curves. The mechanical strength indices included the maximum load (N) and stiffness (N/mm).

### 4.14. Statistical Analysis

The data are presented as means ± SD. Statistical analyses were performed using GraphPad Prism 8.0 software (GraphPad Software, San Diego, CA, USA). Statistical significance was calculated using a two-tailed Student’s *t*-test or one-way analysis of variance (ANOVA). *p* < 0.05 was considered statistically significant. All data were generated from three or more independent experiments.

## Data Availability

Raw mass spectrometry data have been deposited in the iProX database (http://www.iprox.org (Beijing, China)) with the iProX accession number PXD049098.

## Supplementary Materials

The following supporting information can be downloaded at: https://www.mdpi.com/article/10.3390/ijms25168870/s1.
