# Supplementary material for: The Discovery of a Specific CKIP-1 Ligand for the Potential Treatment of Disuse Osteoporosis
Source: Int J Mol Sci. 2024 Aug 15;25(16):8870. doi: 10.3390/ijms25168870 (PMC11354310; doi:10.3390/ijms25168870)
Supplement: Supplementary file 1 [file ijms-25-08870-s001.zip › Supplementary Data.pdf]

# Supplementary Materials for

## The Discovery of a Specific CKIP-1 Ligand for the Potential Treatment of Disuse Osteoporosis

Yange Wei, Bo Wu, Mingqiu Liu and Chun-Ping Cui *

* Correspondence: cui_chunping2000@aliyun.com; Tel.: +86-13671215785

This PDF file includes:

**Materials and methods**

**Characterisation data for C77**

**Experimental for largest scale run for C77**

**The flowchart of DELs screening experimental procedure**

# Supplementary Data

## 1. Materials and methods

### 1.1 Antibodies and reagents

The following antibodies were used: anti-VHL (Santa Cruz, #135657, 1:500, for IF), anti-CKIP-1 (Proteintech, #24883-1-AP, 1:500, for IF), anti-CKIP-1 (Santa Cruz, #376355, 1:500), anti-GAPDH (Santa Cruz, #365062, 1:2000), anti-normal IgG (Santa Cruz, #2003, 1:5000), anti-$\alpha$-Tubulin (Cell Signaling Technology, #2144, 1:1000), anti-Myc (MBL, #M047-3, 1:2000), anti-Flag (MBL, M185-3, 1:2000), anti-HA (MBL, #M180-3, 1:2000), and anti-Multi Ubiquitin (MBL, #D058-3, 1:2000), anti-β-actin (Abclonal, #AC026, 1:2000) and HSP90 (Abclonal, #A5027, 1:2000). Peroxidase-AffiniPure goat anti-rabbit IgG (Jackson, #111-035-003, 1:500) and Peroxidase-AffiniPure goat anti-mouse IgG (Jackson, #115-035-003, 1:500).

The following reagents were used: MG132 (Sigma-Aldrich), Puromycin (MCE, USA), Cycloheximide (CHX, MCE, USA) and Polybrene (Sigma-Aldrich), HaloPROTAC3 (Promega, USA).

### 1.2 Protein half-life assay

For the exogenous CKIP-1 half-life assay, plasmids encoding CKIP-1 and VHL were transfected into HEK293T or BMSCs. After 24 hours, the cells were treated with the protein synthesis inhibitor CHX (100 μg/ml) for specified durations before collection. For the endogenous CKIP-1 half-life assay, BMSCs were treated with CHX and either with or without MG132 (20 μM) for indicated durations before collection. The cells were then lysed and subjected to immunoblotting.

### 1.3 His pull-down by Ni-NTA columns

For the in vitro ubiquitination assay, Flag-CKIP-1, Myc-VHL, and His-Ub, were transfected into HEK293T cells for 24 h. Then, the cells were treated with the proteasome inhibitor MG132 for 8 hours. Cells were collected and lysed under denaturing conditions (buffer A: 6 M guanidine-HCl, 100 mM sodium phosphate, 10 mM imidazole (pH 8.0)) on ice. After sonication for 2 min and centrifugation at 12,000 × g for 15 min, the supernatant was incubated with nickel-nitrilotriacetic acid (Ni-NTA) matrices for 4 h. Then, the His-tag pulldown products were washed sequentially once in buffer A, twice in a buffer A/buffer TI mixture (buffer A: buffer TI =1:3), and once in buffer TI (25 mM Tris-HCl and 20 mM imidazole (pH 6.8)) to purify the polyubiquitinated proteins at room temperature. The polyubiquitinated proteins were separated by SDS-PAGE and subjected to immunoblotting with indicated antibodies.

## 2. Characterisation data for C77

A

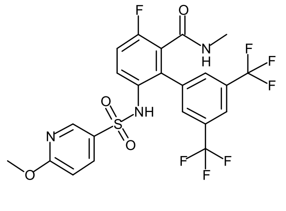


B

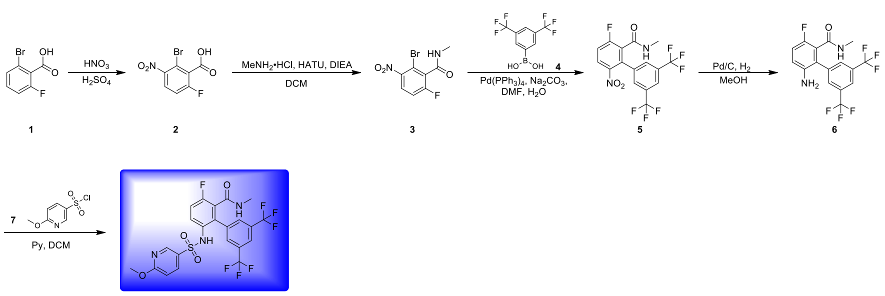


**10172-10051-15-479-0-77**

C

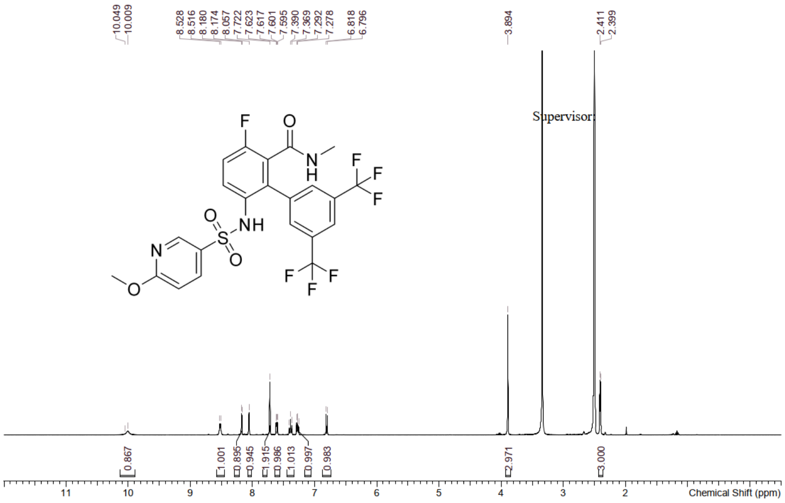


D

# LCMS Report

=====================================

| | |
|---|---|
| Compound ID | : 10172-10051-15-479-0-77 |
| Sample ID | : EC541-518-P1C2 |
| Injection Vol | : 1ul |
| Location | : vial82 |
| Acq Method | : D:\method\5-95AB_1min.lcm |
| Org DataFile | : D:\Data\2021\2107\210723\EC541-518-P1C2.lcd |
| Injection Date | : 7/23/2021 3:28:35 PM |
| Instrument | : CAS-CD-LCMS-D |

Chromatogram

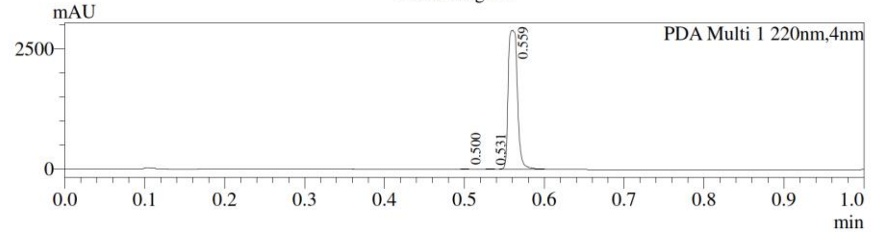

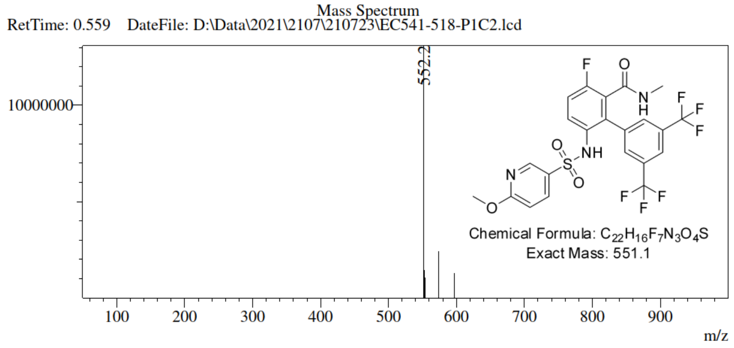


E

# HPLC Report

Compound ID : 10172-10051-15-479-0-77
Sample ID : EC541-518-P1C4
Injection Vol : 1ul
Location : vial23
Acq Method : D:\method\10-80AB_2min.lcm
Org DataFile : D:\DATA\2021\2107\210723\EC541-518-P1C4.lcd
Injection Date : 7/23/2021 4:51:02 PM
Instrument : CAS-CD-HPLC-A

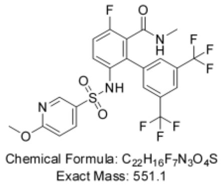


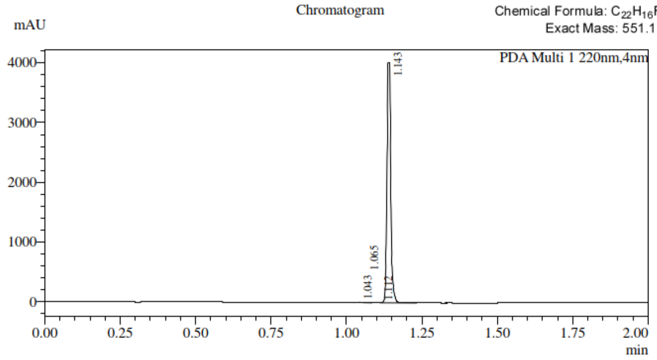


**Integration Result**

PDA Ch1 220nm

| Peak# | Ret. Time | USP Width | Height | Height% | Area | Area% |
|---|---|---|---|---|---|---|
| 1 | 1.043 | 0.020 | 7417 | 0.184 | 5638 | 0.156 |
| 2 | 1.065 | 0.025 | 2731 | 0.068 | 2237 | 0.062 |
| 3 | 1.112 | 0.018 | 4620 | 0.115 | 3144 | 0.087 |
| 4 | 1.143 | 0.019 | 4016206 | 99.634 | 3602595 | 99.695 |
| Total | | | 4030973 | 100.000 | 3613615 | 100.000 |

(A). Chemical structure of C77.

(B). The synthetic route of C77.

(C). The nudear magnetic resonance spectrum of C77.

(D). Liquid Chromatograph Mass Spectrum List Report of C77.

(E) High Performance Liquid Chromatography Report of C77.

**3. Experimental for largest scale run for C77**

*1.) General procedure for preparation of Compound 2 - 2-bromo-6-fluoro-3-nitro-benzoic acid -*

*Notebook Page: EC541-496*

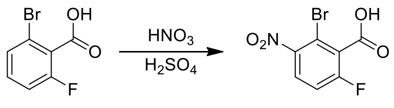


**1** **2**

To a solution of 2-bromo-6-fluoro-benzoic acid (3.00 g, 13.7 mmol, 1.00 eq) in concentrated sulfuric acid (9.0 mL) was added nitric acid (881 mg, 13.7 mmol, 629 uL, 98% purity, 1.00 eq) at 0°C. The mixture was stirred at 25°C for 3 h. The mixture was poured into ice/water (40.0 mL), a large number of solid precipitated out, filtered and the solid was washed with water (20.0 mL), dried under high vacuum to get 2-bromo-6-fluoro-3-nitro-benzoic acid (2.9 g, crude) as a white solid.

*2.) General procedure for preparation of Compound 3 - 2-bromo-6-fluoro-N-met hyl-3-nitro-benzamide - Notebook Page: EC541-498*

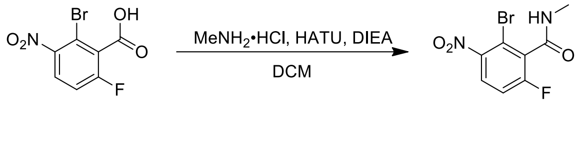


**2** **3**

To a solution of 2-bromo-6-fluoro-3-nitro-benzoic acid (2.90 g, 11.0 mmol, 1.00 eq) in dichloromethane (30.0 mL) was added N,N-diisopropylethylamine (4.26 g, 33.0 mmol, 5.7 mL, 3.00 eq), O-(7-azaben zotriazol-1-yl)-N,N,N,N-tetramethy luroniumhexa fluorophosphate (5.01 g, 13.2 mmol, 1.20 eq) and methanamine; hydrochloride (890 mg, 13.2 mmol, 1.20 eq) at 0°C. The mixture was stirred at 20°C for 3 h. The reaction mixture was diluted with water (50.0 mL) and extracted with dichloromethane (50.0 mL * 2). The combined organic layers were washed with sat. sodium bicarbonate (50.0 mL) and brine (50.0 mL), the collected organics were dried over anhydrous sodium sulfate, filtered and concentrated under reduced pressure to give a residue. The residue was purified by silica gel column chromatography (petroleum ether/ethyl acetate = 3/1 ~ 1/1) to get 2-bromo-6-fluoro-N-methyl-3-nitro-benzamide (680 mg, 2.21 mmol, 20.11% yield) as a yellow solid.

*3.) General procedure for preparation of Compound 5 - 2-[3,5-bis(trifluoromethyl)phenyl] -6-fluoro-N-methyl-3-nitro-benzamide - Notebook Page: EC541-512*

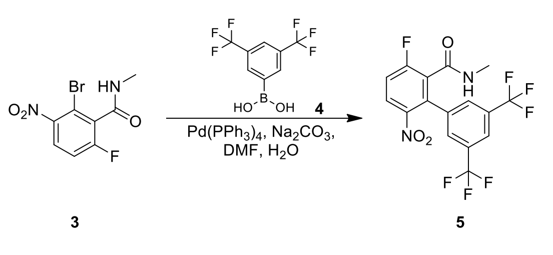


**3** **5**

A mixture of 2-bromo-6-fluoro-N-methyl-3-nitro-benzamide (580 mg, 2.09 mmol, 1.00 eq), [3,5-bis

(trifluoromethyl)phenyl]boronic acid (810 mg, 3.14 mmol, 1.50 eq), tetrakis[triphenylphosphine]palla dium(0) (484 mg, 419 umol, 0.200 eq), sodium carbonate (666 mg, 6.28 mmol, 3.00 eq) in water(0.5 mL) and dimethylformamide (5.5 mL) was degassed and purged with nitrogen for 3 times, and then the mixture was stirred at 90°C for 1 h under nitrogen atmosphere. The mixture was diluted with water (15.0 mL) and extracted with ethyl acetate (15.0 mL * 2), dried over sodium sulfate, filtered and concentrated under reduced pressure. The residue was purified by silica gel column chromatography (petroleum ether/ethyl acetate = 3/1) to get 2-[3,5-bis(trifluoromethyl)phenyl]-6-fluoro-N-methyl-3-nitro-benzamide (618 mg, 1.36 mmol, 64.7% yield) as a white solid.

*4.) General procedure for preparation of Compound 6 - 3-amino-2- [3,5-bis (trifluoromethyl)phenyl]-6-fluoro-N-methyl-benzamide - Notebook Page: EC541-516*

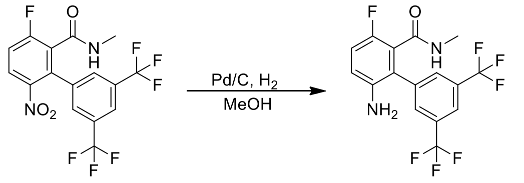


5 → 6

To a solution of 2-[3,5-bis(trifluoromethyl)phenyl]-6-fluoro-N-methyl-3-nitro-benzamide (400 mg, 975 umol, 1.00 eq) in methanol (8.0 mL) was added palladium on carbon (103 mg, 97.5 umol, 10% purity,) under nitrogen atmosphere. The suspension was degassed and purged with hydrogen for 3 times. The mixture was stirred under hydrogen (15 psi) at 25°C for 8 h. The reaction mixture was filtered and the filter was concentrated to get 3-amino-2-[3,5-bis(trifluoromethyl)phenyl]-6-fluoro-N-methyl-benzamide (502 mg, crude) as a brown solid.

*5.) General procedure for preparation of Compound 10172-10051-15-479-0-77 - 2-[3,5-bis(trifluoromethyl)phenyl]-6-fluoro-3-[(6-methoxy-3-pyridyl)sulfonylamino]-N-methyl-benzamide - Notebook Page: EC541-518*

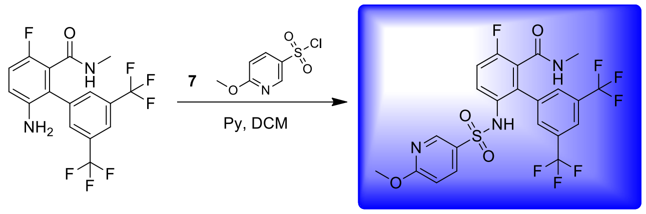


6 → 10172-10051-15-479-0-77

To a mixture of 3-amino-2-[3,5-bis(trifluoromethyl)phenyl]-6-fluoro-N-methyl-benzamide (250 mg, 657 umol, 1.00 eq) and

pyridine (624 mg, 7.89 mmol, 637 uL, 12.0 eq) in dichloromethane (1.50 mL) was added a solution of 6-methoxypyridine-3-sulfonyl chloride (137 mg, 657 umol, 1.00 eq) in dichloromethane (1.5 mL) dropwise at 0°C, then it was stirred at 25°C for 2 h. Additinal 6-methoxy pyridine-3-sulfonyl chloride (137 mg, 657 umol, 1.00 eq) was added, then it was stirred at 25°C for 2 h. The reaction mixture was diluted with water (50.0 mL) and extracted with dichloromethane (50.0 mL * 2). The combined organic layers were washed with saturated sodium bicarbonate solution (50.0 mL) and brine (50.0 mL), dried over anhydrous sodium sulfate, filtered and concentrated under reduced pressure to give a residue. The crude product was purified by prep-HPLC (column: Phenomenex Luna C18 100*30mm*5um; mobile phase: [water(formic acid)- acetonitrile];B%: 42%-72%,8min) to get 2-[3,5-bis(trifluoromethyl)phenyl]-6-fluoro-3-[(6-methoxy-3-pyridyl)sulfonylamino]-N-methyl-benzamide (40.08 mg, 72.2 umol, 11.0% yield) as a white solid.

## 4. The flowchart of DELs screening experimental procedure

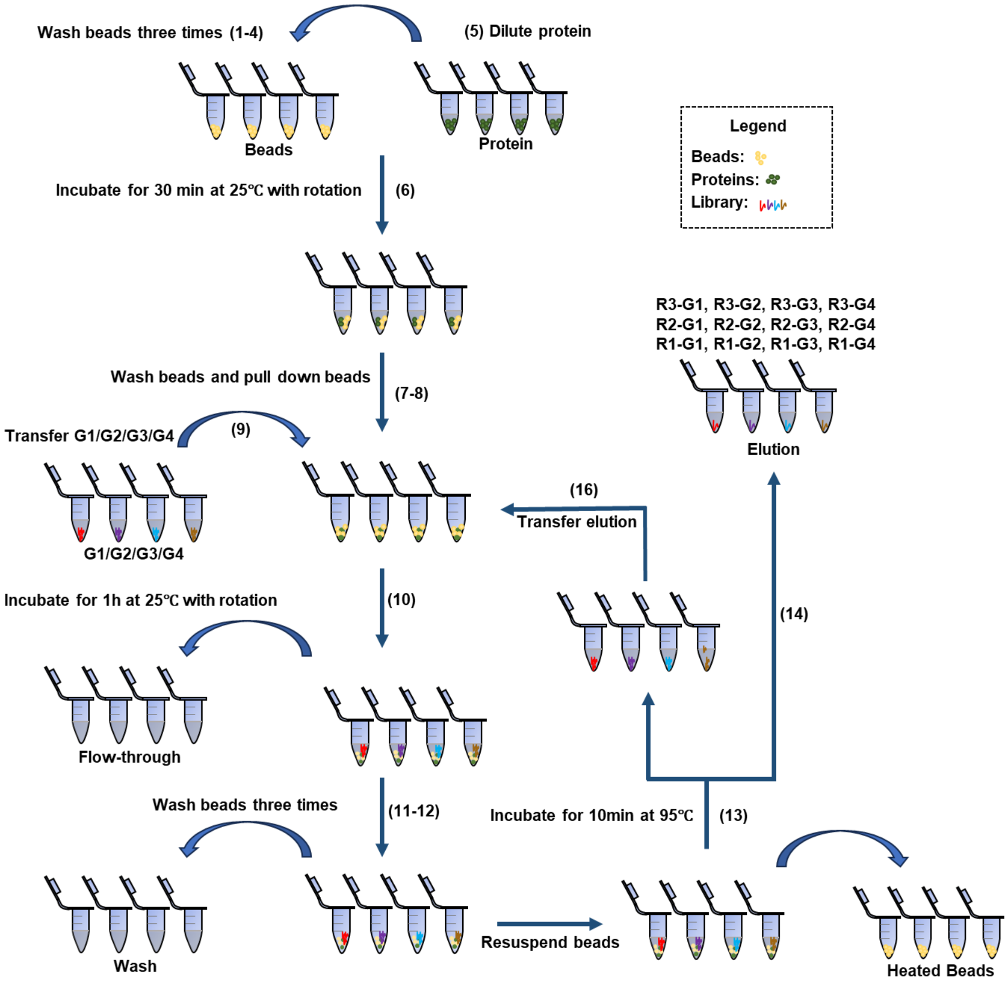

Notes:

1. Fresh immobilized proteins-beads should be used in each round of selection.
2. DEL libraries should be used up for the first round of selection.
3. Repeat step 1-6 to prepare fresh protein-beads matrix for Round 2/3 is step 15.
4. Retain 10μL of 1st round eluate as R1-G1, R1-G2, R1-G3, R1-G4.

Retain 50 μL of 2nd round eluate as R2-G1, R2-G2, R2-G3, R2-G4.

Retain all 3rd round eluate as R3-G1, R3-G2, R3-G3, R3-G4.
