# Supplementary material for: The Discovery of a Specific CKIP-1 Ligand for the Potential Treatment of Disuse Osteoporosis
Source: Int J Mol Sci. 2024 Aug 15;25(16):8870. doi: 10.3390/ijms25168870 (PMC11354310; doi:10.3390/ijms25168870)
Supplement: Supplementary file 1 [file ijms-25-08870-s001.zip › Supplementary Figures and Table.pdf]

Supplementary Materials for

The Discovery of a Specific CKIP-1 Ligand for the Potential Treatment of Disuse Osteoporosis

Yange Wei, Bo Wu, Mingqiu Liu and Chun-Ping Cui *

* Correspondence: cui_chunping2000@aliyun.com; Tel.: +86-13671215785

This PDF file includes:

Figures. S1 to S6
Table S1

Supplementary Figure S1

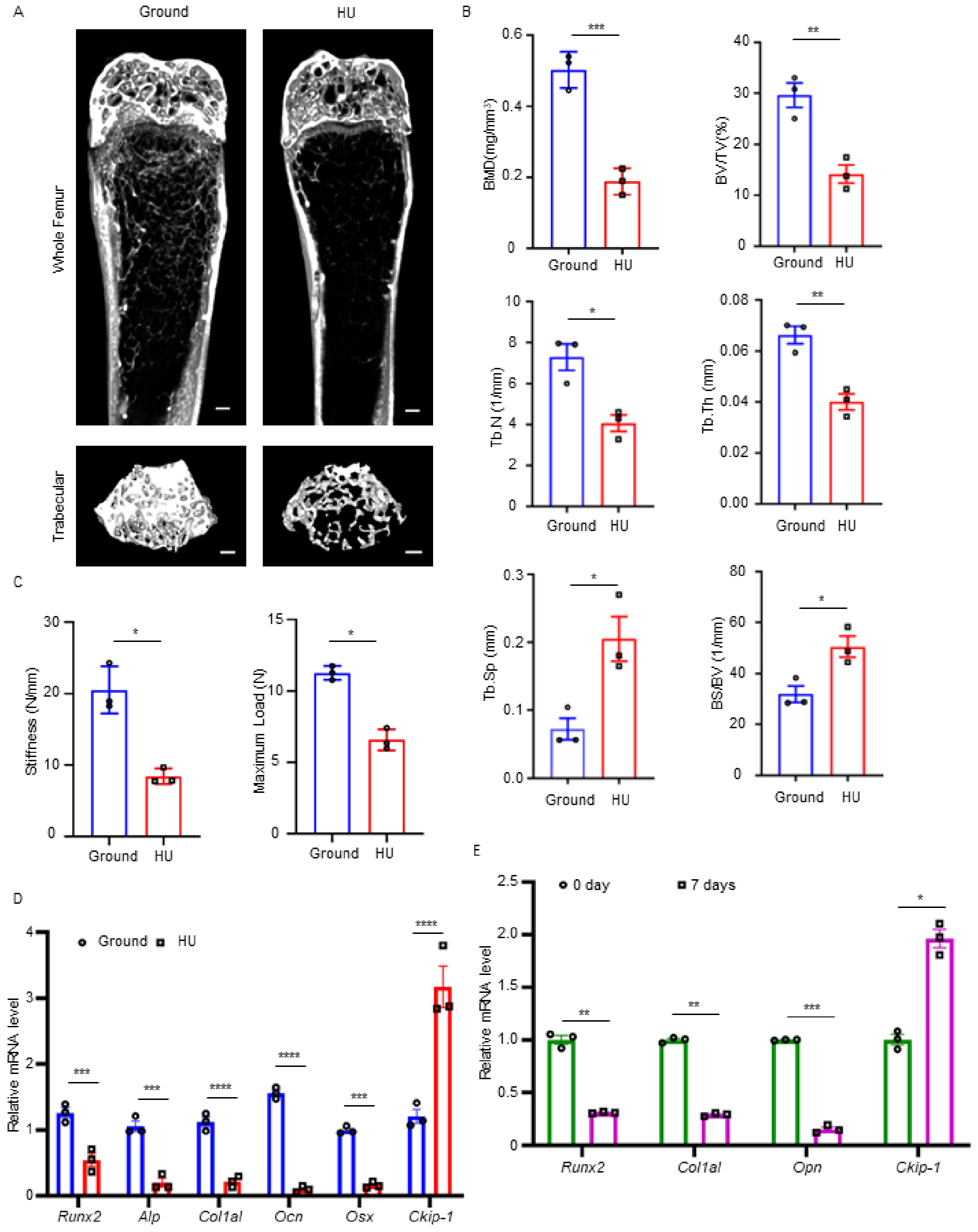

Figure S1. Construction of microgravity model.

(A) Representative micro-CT images of whole femoral (top) and trabecular (bottom) bones from 3-months-old male ground and HU mice. n=3 per group. Scale bars, 0.5 mm.

(B) Histomorphometric analysis of distal femurs from (A), includ-ing bone mass density (BMD), bone volume per tissue volume (BV/TV), trabecular number (Tb.N), trabecular thickness (Tb.Th), trabecular spacing (Tb.Sp), and bone surface per bone vol-ume (BS/BV).

(C) Bone mechanical property was detected by three-point bending test, including stiffness, and maximum load.

(D) Quantitative RT-PCR analysis of osteogenesis genes (*Runx2*, *Alp*, *Col1a1*, *Ocn* and *Osx*) and *Ckip-1*

mRNA levels in BMSCs from 3-months-old male ground and HU mice. n =3 per group.

(E) Quantitative RT-PCR analysis of osteogenesis genes (*Runx2*, *Col1a1*, and *Opn*) and *Ckip-1* mRNA levels in BMSCs after RCCS.

All data shown as mean± s.d. **p* <0.05, ***p* <0.01, ****p* <0.001, *****p* <0.0001.

Supplementary Figure S2

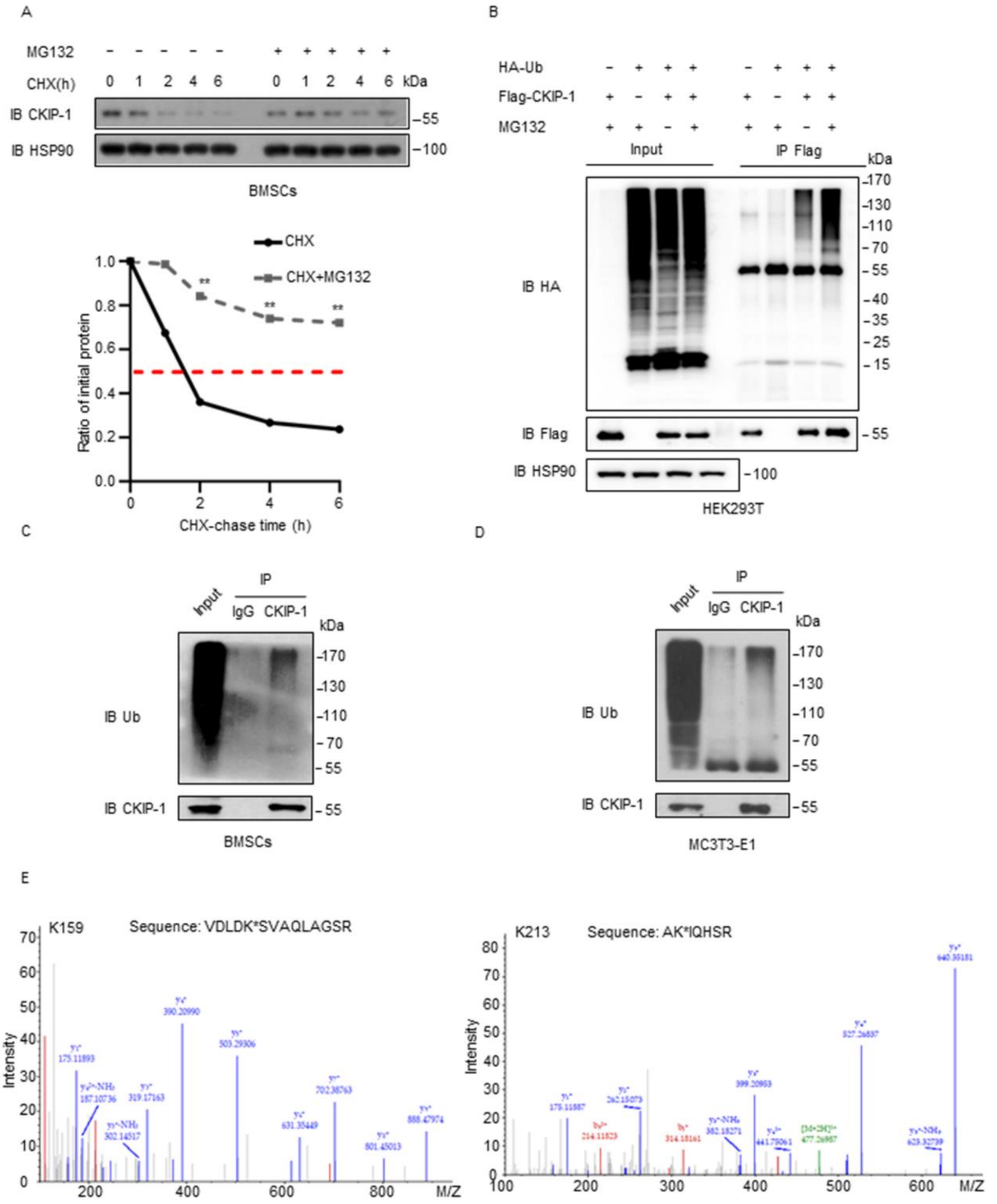

Figure S2. CKIP-1 can be ubiquitinated in osteoblasts.

(A) BMSCs cells were treated with CHX and with or without MG132 (20 μM) for the indicated times. Whole-cell lysates were prepared and analyzed by immunoblotting. The graph shows quantification of relative CKIP-1 levels. The results are shown as mean ± s.d. ***p* <0.01.

(B) HEK293T cells were transfected with plasmids that expressed Flag-tagged CKIP-1 and HA-tagged

ubiquitin. 24 hours after transfection, cells were treated with or without MG132 (20 μM) for 8 h before cell lysis.

(C) CKIP-1 in osteoblasts BMSCs were immunoblotted with anti-ubiquitin.

(D) CKIP-1 in pre-osteoblasts MC3T3-E1 were immunoblotted with anti-ubiquitin.

(E) MS spectrogram of the CKIP-1 ubiquitination sites K159 and K213. n =3 independent experiments.

Supplementary Figure S3

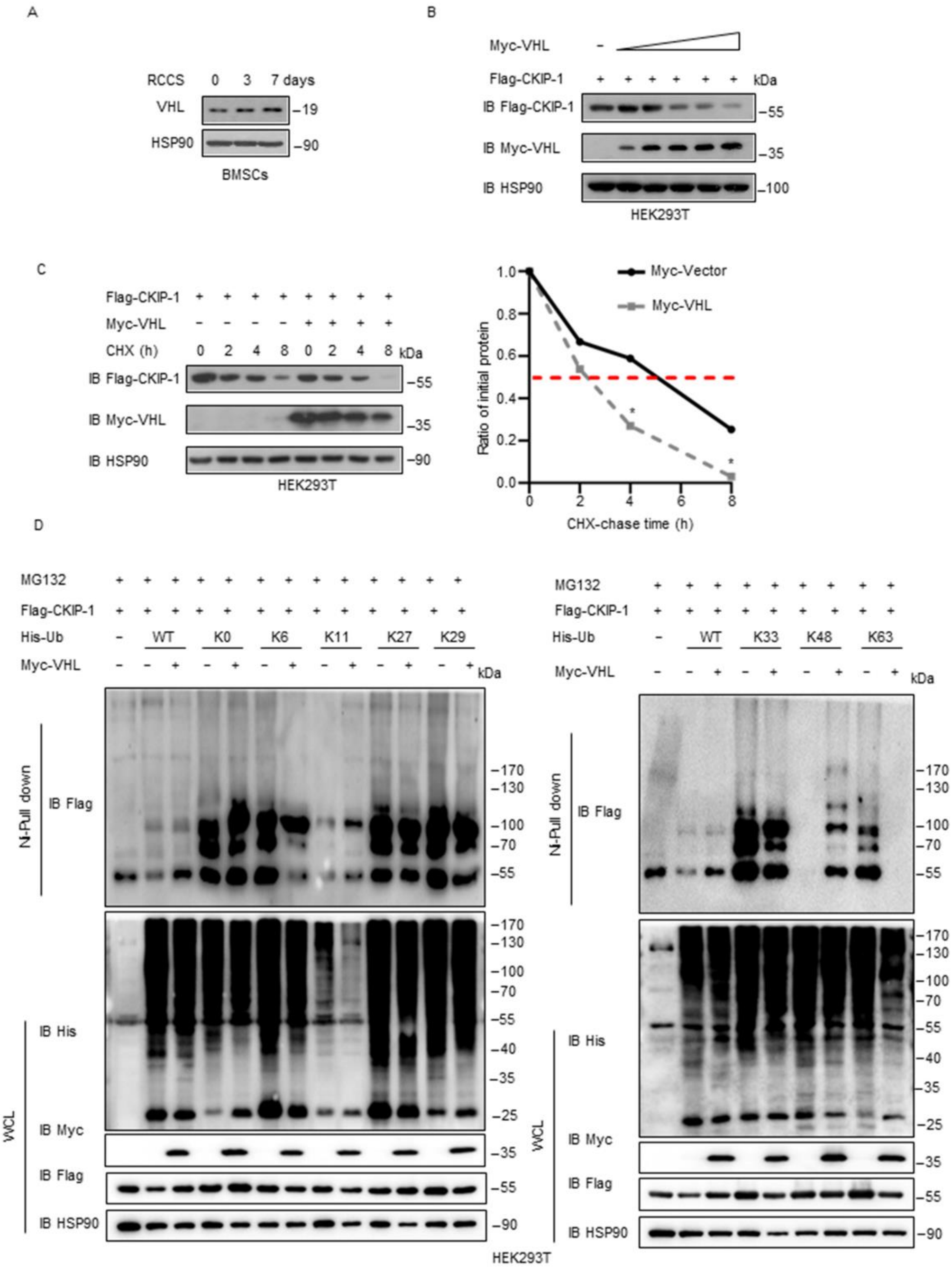

Figure S3. VHL promotes ubiquitylation of CKIP-1.

(A) Immunoblot analysis of VHL levels in BMSCs were cultured in RCCS for 3 days or 7 days.

(B) HEK293T cells were transfected for 24 h with Flag-tagged CKIP-1 and increasing amounts of Myc-tagged VHL plasmids. The indicated proteins were detected by immunoblotting with indicated

antibodies. Results are shown as mean ± s.d. *p <0.05.

(C) HEK293T cells were transfected for 24 h with Flag-tagged CKIP-1 alone or together with Myc-tagged VHL plasmids, and then treated with CHX for indicated time period. The indicated proteins were detected by immunoblotting with indicated antibodies. Quantification of relative CKIP-1 levels is shown.

(D) His-Ub or mutant Ub was co-transfected together with indicated Flag-tagged CKIP-1, Myc-tagged VHL into HEK293T cells. Cells were treated with MG132 (20 μM) for 8 h before collection. Then His-Ub was pulled down by Ni-NTA and immunoblotted with indicated antibodies. n =3 independent experiments.

Supplementary Table S1: Mouse qPCR primers

**Name**	**5' primer**	**3' primer**
Gapdh	TGCACCACCAACTGCTTAG	GGATGCAGGGATGATGTTC
Ckip-1	AGGAATCCCTACCCCCTGTC	GCAGCTCCTGAGTCTTCTCC
Ocn	CTCACAGATGCCAAGCCCA	CAAGGTAGCGCCGGAGTCT
Osx	GCAAGGCTTCGCATCTGAAA	AACTTCTTCTCCCGGGTGTGA
Alp	ATCTTTGGTCTGGCTCCCATG	TTTCCCGTTCACCGTCCAC
Col1a1	AGACATGTTCAGCTTTGTGGAC	GCAGCTGACTTCAGGGATG
Opn	ACACTTTCACTCCAATCGTCC	TGCCCTTTCCGTTGTTGTCC
Runx2	CCCAGCCACCTTTACCTACA	TATGGAGTGCTGCTGGTCTG
